# Journal of conservative dentistry is now PUBMED indexed

**DOI:** 10.4103/0972-0707.62628

**Published:** 2010

**Authors:** Velayutham Gopikrishna, Krithika Datta, S Nandini

**Affiliations:** 1 Journal of Conservative Dentistry, Department of Conservative Dentistry, Meenakshi Ammal Dental College, Alapakkam Main Road, Maduravoyal, Chennai – 600 095, Tamil Nadu, India. E-mail: editor@jcd.org.in; 2 Journal of Conservative Dentistry, Department of Conservative Dentistry, Meenakshi Ammal Dental College, Alapakkam Main Road, Maduravoyal, Chennai – 600 095, Tamil Nadu, India; 3 Journal of Conservative Dentistry, Department of Conservative Dentistry, Meenakshi Ammal Dental College, Alapakkam Main Road, Maduravoyal, Chennai – 600 095, Tamil Nadu, India

The mission of a journal is not only to serve as a portal for the dissemination of research knowledge, but also to ensure that vital information reaches as many students, teachers, and researchers as possible. Indexing of a journal by reputed agencies helps one achieve this task. The indexing of a journal in PUBMED is a significant milestone in the journey of the Journal of Conservative Dentistry to be a journal of significance. It will now be possible for other nations to learn and understand the nature of restorative and endodontic research practice emanating from our country.

This achievement for our journal could not have been possible without the support and guidance of many. *Medknow Publication* — an enterprise with a dedicated team — helped us immensely and opened up to us the world of online handling of articles. Access through cyberspace has opened many avenues to authors who want to publish relevant articles in the Journal of Conservative Dentistry, thereby effectively reducing the turn-around time between submission to approval.

It would have been impossible to handle this huge inflow of submissions without the diligent review process done by our esteemed panel of *Peer-reviewers*. It is indeed their wealth of knowledge gathered by years of experience that has resulted in filtering out the best in every article.

If not for anybody, this achievement is a toast to the mind that sparks research questions and works relentlessly toward finding the answers, gathers facts and figures, and presents them in a manuscript. We acknowledge and appreciate the contribution of every *Author* in driving the Journal of Conservative Dentistry to a better platform.

Research completed and not published is the same as research not done at all. It is not only important to pursue a research question with enthusiasm and dedication, but it is equally important to have the perseverance to publish the research findings. This serves two purposes; to share something that was done and to save time and resource for others in researching the same question. The biggest asset of our Indian fraternity is the volume of specialists graduating every year. As compared to 60 dental schools in USA, 10 in Australia, and 17 in UK, there are over 275 dental colleges in India catering to the field of Conservative Dentistry and Endodontics. One can imagine the amount of research activity generated through these institutions. If not for anything, such voluminous research studies should reflect in the number of publications from our country. The road to make such a difference globally is to ask simple, but honest research questions. We now have a platform to showcase our research capabilities to the world. The Journal of Conservative Dentistry will be the portal that will propel our speciality to many more milestones in the future. Godspeed!

